# The influence of prolonged aerobic exercise on cardiac, muscular, and renal biomarkers in trained individuals with obesity

**DOI:** 10.1007/s00421-024-05697-8

**Published:** 2025-01-09

**Authors:** M. D’Alleva, J. M. Sanz, N. Giovanelli, F. Graniero, L. Mari, R. Spaggiari, D. Sergi, S. Ghisellini, A. Passaro, S. Lazzer

**Affiliations:** 1 Department of Medicine, University of Udine, P. le Kolbe 4 – 33100, Udine, Italy; 2 School of Sport Sciences, University of Udine, Udine, Italy; 3 Department of Chemical and Pharmaceutical and Agricultural Sciences, University of Ferrara, Ferrara, Italy; 4 Physical Exercise Prescription Center, Azienda Sanitaria Universitaria Friuli Centrale, Gemona del Friuli, Udine, Italy; 5 Department of Translational Medicine, University of Ferrara, Ferrara, Italy; 6 Biochemical Analysis Laboratory - Clinics and Microbiology, University Hospital of Ferrara, Ferrara, Italy

**Keywords:** Obesity, Cardiorespiratory fitness, Half-marathon, Marathon, Cardiac damage, Muscular damage, Renal injury

## Abstract

**Purpose:**

The aim of this study was to investigate the influence of prolonged aerobic exercise on cardiac, muscular and renal inflammatory markers in a group of trained obese men.

**Methods:**

Seventeen men (aged 40 ± 6 years; body mass index [BMI] 31.3 ± 2.8 kg m^−2^, maximal oxygen uptake [V’O_2_max] 41.5 ± 5.6 ml kg^−1^ min^−1^) ran a half, 30 km, or full marathon. Troponin I (cTnI), the n-terminal creatine kinase-myocardial band (CK-MB), pro b-type natriuretic peptide (NT-proBNP), lactate dehydrogenase (LDH), myoglobin, creatinine (CREA) and the estimated glomerular filtration rate (eGFR) were measured before (T0), immediately after (T1) and 3 days after the race (T2).

**Results:**

The concentrations of cTnI, myoglobin, LDH, CK-MB and CREA significantly increased (*P* < 0.05), whereas eGRF decreased at T1 (*P* < 0.05). All the above parameters returned to baseline at T2, except for eGFR, which remained lower than that at T0 (*P* < 0.05). A positive association was observed between ΔCK-MB (%) and the time spent in Zone 3 during the race (*R* = 0.686, *P* = 0.014). The Δmyoglobin (%) was positively correlated with race time, race mean speed and time in Zone 3 (*R* = 0.574–0.862, *P* < 0.05). The ∆CREA values were moderately correlated with the race mean HR_MAX_ (%) and time spent in Zone 3 (%) (*R* = 0.514–0.610; *P* = 0.05). The ∆eGRF values were moderately inversely correlated with the time spent in Zone 3 (%) (*R* =  – 0.627; *P* < 0.05).

**Conclusion:**

Changes in cardiac, muscular and renal inflammatory markers in trained men with obesity are consistent with those described in the literature in normal-weight individuals. Finally, running parameters, such as running time, average running intensity and time in Zone 3 appear to be responsible for the changes in cardiac, muscular and renal function markers after long-distance running.

## Introduction

Endurance training (i.e. walking, cycling, or running) is an important lifestyle intervention to promote a negative energy balance and elicit body weight loss in overweight and individuals with obesity (Petridou et al. 2019). Furthermore, endurance training improves fitness parameters such as maximal oxygen consumption (V’O_2_max) (Su et al. 2019) and reduces the risk of all-cause and cardiovascular mortality (Gaesser and Angadi 2021). Moreover, the number of people with obesity who participate in running events is increasing (Vincent et al. 2020). In addition, including challenging sports performance at the end of training programmes seems to be useful for increasing the motivation and adherence of participants in training (D’Alleva et al. 2023; Keytsman et al. 2019).

Although endurance training is associated with numerous health benefits, long-distance running competitions such as half-marathons or marathons can be physically demanding and induce changes in cardiac, muscular and renal function serum biomarkers (Shin et al. 2016; Bernat-Adell et al. 2019, 2020). Indeed, previous studies have shown an increase in biomarkers of cardiac damage (i.e. cardiac troponin I (cTnI), and N-terminal proB-type natriuretic peptide (Pro-BNP)) above upper reference limits (URL), which are still elevated 24–72 h after prolonged endurance exercise, without clinical symptoms of cardiac disease (Rubio-Arias et al. 2021; Traiperm et al. 2021). This is because prolonged endurance exercise (i.e. over 90 min) requires high cardiac output and increased heart rate and blood pressure, arising from greater myocardial workload (Rubio-Arias et al. 2021; Aengevaeren et al. 2021). Moreover, during endurance running, the lower limbs absorb 1.5 to 3 times the runner’s body mass (BM) at each step over a long period of time (Lieberman et al. 2010), leading to an increase in the serum levels of markers of acute muscle damage, including lactate dehydrogenase (LDH) and myoglobin (Shin et al. 2016; Bernat-Adell et al. 2019). Similarly, prolonged and strenuous aerobic exercise causes endothelial dysfunction, which can trigger the renal changes observed after marathon running (Panizo González et al. 2019) and is marked by a decrease in the estimated glomerular filtration rate (eGFR) and/or an increase in the serum creatinine (CREA) level (Shin et al. 2016; Poussel et al. 2020). These parameters generally revert to physiological values 24–48 h after the running event (Panizo González et al. 2019). However, the determinants leading to increased serum levels of markers of cardiac, muscular and renal damage are still unclear.

Eijsvogels et al. (2012) reported that cTnI values measured before and immediately after a single walk (i.e. 30–50 km) did not differ among lean, overweight and obese participants, and linear regression analysis revealed that exercise intensity, expressed as a percentage of maximal heart rate (HRmax), age and sex, but not body mass index (BMI), fat percentage or waist circumference, was significantly correlated with post-exercise cTnI values (Eijsvogels et al. 2012). The latter was also confirmed by Martínez-Navarro et al. (2020a, b), who reported that high-sensitivity cardiac troponin T (cTnT) after a marathon in recreational runners correlated positively only with the mean heart rate (HR) of the marathon, expressed as bpm or percentage of HRmax, with no significant correlations being detected between cTnT and body composition or cardiorespiratory parameters (Martínez-Navarro et al. 2020b). Instead, Bernat-Adell et al. (2019) reported that after a marathon, only higher energy expenditure was positively correlated with blood levels of LDH and creatine kinase (CK) and that cTnT and race duration were negatively correlated with the release of LDH and cTnT (Bernat-Adell et al. 2019). However, to our knowledge, no studies to date have investigated the changes in cardiac, muscular, and renal function in a group of trained overweight or obese individuals after completing long-distance exercise. Moreover, only a few studies have analysed in detail the relationship between changes in circulating cardiac, muscular and renal parameters and baseline anthropometric characteristics, physical capacities and training parameters of the participants measured during running competition (Bernat-Adell et al. 2019; Martínez-Navarro et al. 2020b).

Thus, the aims of the present study were (i) to determine the effects of a running event lasting more than 90 min (i.e. a half-marathon, 30-km race, or marathon) on cardiac, muscular and renal serum biomarkers in healthy males with overweight or obesity and (ii) to investigate the relationship between the changes in the above parameters after the race and the participants’ baseline characteristics (i.e. body composition and physical capacities) and marathon-related parameters.

## Materials and methods

### Subjects

Seventeen healthy males with obesity (aged 40 ± 6 years; BMI 31.3 ± 2.8 kg m^−2^) were included in this study (D’Alleva et al., 2023). Eight participants were overweight with a BMI between 25 and 30 kg m^−2^ (29.1 ± 0.4 kg m^−2^), whereas nine participants were categorized as first-degree obese because they had a BMI between 30 and 35 kg m^−2^ (32.5 ± 1.7 kg m^−2^).

The recruitment was conducted with a survey that was disseminated (by local press and online) with the collaboration of the Communication Office of the Department of Medicine of the University of Udine, of the office “Uniud Sostenibile” of the University, and the local club FIAB Udine—aBcitUdine. The survey investigated commuting habits, lifestyles and levels of physical activity. All the volunteers took part in a 24-week training study (see the experimental design for more details). All participants had a complete medical history and underwent physical and nutritional assessments, described in detail in the following sections. None of the subjects had cardiovascular, respiratory, neurological, skeletal, metabolic or endocrine diseases, nor did they take medications regularly or drugs known to affect energy metabolism. The baseline characteristics of the participants are presented in Tables 1, 2, 3 and 4.

**Table 1:** Anthropometric characteristics of the participants measured before (T0) the running competition

	All (*N* = 17)
Age (years)	40.6 ± 6.4
Stature (m)	1.76 ± 0.06
BM (kg)	96.2 ± 11.3
BMI (kg m^−2^)	31.3 ± 2.8
FFM (kg)	63.9 ± 6.2
FM (kg)	31.8 ± 7.0
FFM (%)	67.0 ± 4.3
FM (%)	33.0 ± 4.3

All values are presented as mean ± standard deviation

*BM* body mass, *BMI* body mass index, *FFM* fat-free mass, *FM* fat mass

**Table 2:** Physiological parameters of the participants measured before (T0) the running competition

	All (*N* = 17)
*Maximal oxygen uptake*	
V’O_2_(L min^−1^)	3.98 ± 0.50
V’O_2_(ml kg^−1^ min^−1^)	41.5 ± 5.0
HRmax (bpm)	174 ± 8
RER max	1.12 ± 0.05
Speed (km h^−1^)	13.2 ± 1.6
*Respiratory compensation point*	
V’O_2_(L min^−1^)	3.52 ± 0.46
V'O_2_(ml kg^−1^ min^−1^)	37.0 ± 5.3
V’O_2_max (%)	87.5 ± 5.5
HR (bpm)	163 ± 9
HRmax (%)	92.6 ± 3.9
RER	1.02 ± 0.05
Speed (km h^−1^)	11.6 ± 1.6
*Gas exchange threshold*	
V’O_2_(L min^−1^)	2,99 ± 0.38
V’O_2_(ml kg^−1^ min^−1^)	31.8 ± 5.1
V’O_2_max (%)	70.0 ± 12.5
HR (bpm)	146 ± 10
HRmax (%)	81.0 ± 7.1
RER	0.91 ± 0.06
Speed (km h^−1^)	10.0 ± 1.50

All values are presented as mean ± standard deviation

V’O_2_: oxygen consumption, *HR* heart rate, *RER* respiratory exchange ratio, V’O_2_ max (%): percentage of maximal oxygen uptake, *HRmax* (%): percentage of heart rate max

### Experimental design

The study was approved by the Ethics Committee of the Friuli-Venezia-Giulia Region (Italy) (protocol number 1764). All participants were informed about the protocol and aim of the study and provided written informed consent. Enrolled subjects participated in a running challenge after a 24-week training intervention conducted from November 2021 to May 2022 (D’Alleva et al. 2023). Briefly, during the 24-week training programme, participants were randomly assigned to two intervention groups: polarized training (POL) or threshold training (THR). Both training interventions were divided into three 8-week macrocycles following the 3 + 1 mesocycle scheme (i.e. 3 weeks of increased load and 1 week of recovery). In both groups, the training load (TL) increased by ~ 30% between the first and second 8-week macrocycles and by ~ 10% between the second and third 8-week macrocycles. During each recovery week, the TL was reduced by 30%. Considering the concept of training intensity distribution (TID) (Seiler 2010), the POL group spent 91.0 ± 2.4% of the time at low intensity (i.e. below the gas exchange threshold, GET), and the remainder of the training was above the GET during the 24-week training interventions. In contrast, the THR group spent 71.3 ± 9.6% of the time weekly at low intensity and the remaining percentage of exercise above the GET (Campos et al. 2022). One week before the running event (T0), anthropometric characteristics, body composition analysis and a graded exercise test (GRAD) for physical capacity evaluation (i.e. ventilatory thresholds and V’O_2_max) were performed. All tests were performed under medical supervision. Blood samples were collected before the race (T0), immediately after the end of the race (T1) and 72 h after the race (T2).

### Running challenge

The running challenge was a half-marathon, a 30-km run, or a marathon, depending on the level of fitness reached by the participants at the end of the previous training (D’Alleva et al. 2023). The participants ate breakfast 3 h before the race according to their habits. The challenge began at 10 a.m. and included a 10.2-km lap to be repeated according to the distance to be achieved on flat terrain in a city circuit (Buja, Udine, Italy) (average temperature of 18 °C and average relative humidity of 60%). For safety reasons, we set up refreshment stations along the circuit with water, mineral salts and fruit so that participants could eat and drink enough during the race without being registered. The challenge was conducted under medical supervision. During the challenge, HR, the participant’s speed and running time were monitored using their Garmin (Garmin HRMrun, Olathe, USA) or Polar (Polar H10, Finland) chest straps connected to their respective watches. After the competition, all participants uploaded their own data onto an online training diary, Polar Flow (Polar Electro Oy, Finland) or Gamin Connect (Garmin, Olathe, USA). HR and speed during the marathon were reported in absolute values and as percentages (%) of HR and speed at the gas exchange threshold (GET), respiratory compensation point (RCP) and V’O_2_max calculated during the GRAD test at T0. In addition, the time spent in the three physiological zones (e.g., in both absolute values and %) was calculated for each participant, using the HR at GET, RCP and V’O_2_max (Seiler 2010): Zone 1 (Z1), for intensities below GET; Zone 2 (Z2), for intensities between GET and RCP; and Zone 3 (Z3), for intensities above RCP. Moreover, the total training load (TL) of the race was quantified with the training impulse (TRIMP), modified by Lucía et al. (2003) (luTRIMP_HR_), where each zone has a weighting factor multiplied by the time spent in that zone (Foster et al. 2001).

### Anthropometric characteristics and body composition

BM was measured using a manual weighing scale (Seca 709, Hamburg, 165 Germany) with the subject dressed only in light underwear and without shoes. A wall-mounted height board was used to measure height. Body composition was measured using bioelectrical impedance (BIA, Human IM Plus; DS 171 Dietosystem, Milan, Italy). Fat mass (FM) and fat-free mass (FFM) values were determined using the equations described by Gray et al. (1989).

### Graded exercise test (GRAD)

To determine V’O_2_max, HRmax, and ventilatory thresholds, participants performed a graded exercise test on a 400-m track (Gemona del Friuli, Udine, Italy) under medical supervision. A collaborator with a bike set the pace and the runners were instructed to follow the bike. The duration of each step was one minute, and the speed was increased by 0.5 km/h every minute until volitional exhaustion. Oxygen uptake (V’O_2_), carbon dioxide production (V’CO_2_) and HR were measured during this test using a portable metabolic device (K5; Cosmed, Roma, Italy) and a chest strap (Garmin HRMrun, Olathe, USA), respectively. We calibrated the volume and gas analysers before each test using a 3-L calibration syringe and calibration gas (16.00% O_2_ and 5.00% C’O_2_), respectively. We determined the GET and RCP using the V-slope method (Beaver et al. 1986). V’O_2_max was calculated as the average 30-s V’O_2_ according to previously established criteria (Howley et al. 1995): (i) plateau of V’O_2_ (i.e. increase < 150 ml min − 1), (ii) respiratory exchange ratio (RER) > 1.1, and (iii) ≥ 90% of the theoretical maximal heart rate.

### Blood sampling and analysis

The blood samples of fasted participants were centrifuged at 1600 × *g* for 15 min, and serum or EDTA-plasma was aliquoted and stored at -80 °C until use. The EDTA-plasma Pro-BNP and serum CREA, cTnI, CK-MB, LDH and myoglobin were measured at the Clinical Pathology Laboratory of the S. Anna University Hospital (Ferrara, Italy) using standard methods. The reference values for these biochemical parameters are shown in Table 4. The eGFR was estimated using the Chronic Kidney Disease Epidemiology Collaboration (CKD-EPI) equation (Levey et al. 2009):

$$\begin{gathered} eGFR = 141 * {\text{min}} \left( {{\text{serum}} {\text{creatinine}}/{\text{kappa}}, 1} \right){\text{alpha}} \hfill \\ \quad \quad \quad \times {\kern 1pt} \;{\text{max}}\left( {{\text{serum creatinine}}/{\text{kappa}}, 1} \right) - 1.209 \times 0.993{\text{age}} \times {\text{sex}} \times {\text{race}}{.} \hfill \\ \end{gathered}$$

For females: sex = 1.018; alpha =  – 0.329; kappa = 0.7. For males: sex = 1; alpha =  – 0.411; kappa = 0.9, For the Caucasian race, race = 1.

### Statistical analysis

The sample size was calculated a priori (G-Power software, v. 3.1.9.2, University of Kiel, Kiel, Germany), with 8 participants of similar age and marathon finishing time as our volunteers. The effect size f of 1.92 was determined considering the difference in cTnI and LDH before and after training, with an alpha error of 0.05 and a statistical power of 80% (Bernat-Adell et al. 2019; Traiperm et al. 2021) When analysing the inflammatory markers, all participants were considered together, as we could not detect any differences before and after the race across the three conditions based on our partial data. The data were analysed via GraphPad Prism (version 10.2.0), with significance set at *P* < 0.05. The Shapiro–Wilk test was used to assess the normality of the data. The results obtained are expressed as the means and standard deviations (SDs) for normally distributed data, whereas in the case of a nonnormal distribution, the data are expressed as medians and interquartile ranges. Sphericity was verified by Mauchly’s test. ANOVA for repeated measures was used to assess differences in CREA, eGFR and BM across the three measurement times of T0, T1 and T2. A Greenhouse–Geisser correction was used for violations of the sphericity assumption. The Friedman test was used to assess differences in nonnormally distributed parameters (cTnI, CK-MB, Pro-BNP, and myoglobin) across T0, T1 and T2. Pearson or Spearman correlations were used to analyse the possible relationships between baseline anthropometric characteristics, physical capacity, and running-related variables with changes (Δ) in cardiac, muscular and renal biomarkers. The ∆ values were calculated as follows: Δ = [(T1-T0)/T0]. The correlation was classified as low (*r* = 0.30–0.50), moderate (r = 0.50–0.70), high or very high (*r* = 0.70–1.00) (Atkinson and Nevill 1998). Finally, effect sizes comparing pre–post changes in blood parameters were calculated as the corrected effect size (ES) (Lakens 2013). An ES < 0.20 was considered small, < 0.50 medium and > 0.50 large (Cohen 1988).

## Results

### Participant characteristics

The anthropometric parameters of the 17 participants are shown in Table 1. The physiological characteristics related to V’O_2_max, RCP and GET are shown in Table 2.

**Fig. 1:**
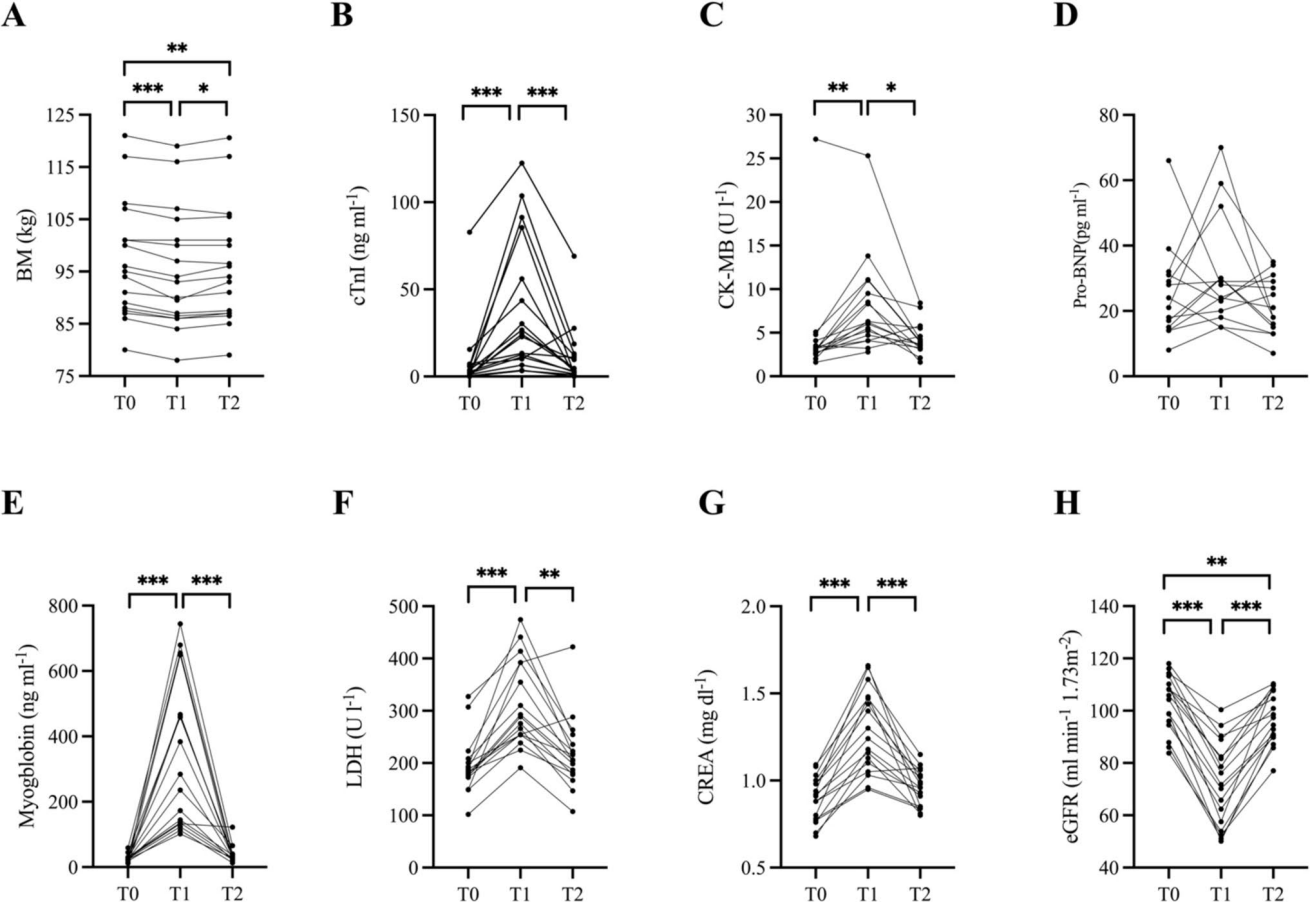
Individual values of BM (**A**), cTnI (**B**), CK-MB (**C**), Pro-BNP (**D**), myoglobin (**E**), LDH (**F**), CREA (**G**), and eGRF (**H**) measured before (T0), immediately after (T1) and 72 h after (T2) the running competition. ANOVA for repeated measure was used to assess the differences in BM, CREA and eGRF between T0, T1 and T2. Friedman test was used to assess the differences in cTnI, Pro-BNP, myoglobin, LDH and CK-MB between T0, T1 and T2. BM: body mass, CREA: creatinine, eGFR: estimated glomerular filtration rate, cTnI: cardiac troponin I, Pro-BNP: N-terminal proB-type natriuretic peptide, *LDH* lactate dehydrogenase, *CK-MB* creatine kinase-myocardial band. *Significantly different *P* < 0.05. ** ignificantly different *P* < 0.01. ***Significantly different *P* < *0.001*

At T1, the BM was significantly reduced by  – 0.94 ± 1.07 kg (*P* < 0.001, ES. 0.10 *small*) (Fig. 1, Panel A). At T2, the BM increased compared with that at T1 (*P* < 0.014, ES. 0.07 *small*) and was  – 0.01 ± 0.01 kg lower than that at T0 (*P* = 0.002, ES 0.07 *small*) (Fig. 1 Panel A).

### Training characteristics

Eight participants completed the half-marathon distance at 2:47:50 ± 0:7:44 h:m:s, range [2:38:57–3:01:06 h:m:s]. Three participants completed the 30-km run at 4:03:04 ± 0:42:03 h:m:s, range [3:35:59–4:51:30 h:m:s], and six participants completed the marathon distance at 4:19:46 ± 0:40:50 h:m:s, range [3:28:14–5:18:19 h:m:s]. The average intensity for the half-marathon was 91.0 ± 4.3% of the HRmax and 63.2 ± 3.5% of the maximal velocity (V_MAX_). For the 30-km run, the average intensity was 83.3 ± 5.3% of HRmax and 55.1 ± 6.2% of V_MAX_. Finally, during the marathon test, the average intensity was 86.0 ± 11.0% of the HRmax and 66.0 ± 5.3% of the V_MAX_. Further details on the training runs can be found in Table 3.

**Table 3:** Exercise variables of the participants measured during the running competition

	All (*N* = 17)		
	42.195 km (*N* = 6)	30 km (*N* = 3)	21.097 km (*N* = 9)
Time (hh:mm:ss)	04:19:46 ±00.4051	04:03:04 ± 00.42:03	02:47:50 ± 00:07:44
Race mean HR (bpm)	154 ± 3	153 ± 8	148 ± 17
Race mean HR_MAX_ (%)	91.0 ± 4.3	83.3 ± 5.3	86.0 ± 11.0
Race mean HR_RCP_ (%)	97.3 ± 3.4	88.9 ± 9.6	91.3 ± 10.8
Race mean HR_GET_ (%)	107.1 ± 5.7	100.2 ± 11.5	101.5 ± 6.0
Race mean speed (km h^−1^)	9.9 ± 1.6	7.5 ± 1.2	7.6 ± 0.3
Race mean speed V_MAX_ (%)	66.0 ± 5.3	55.1 ± 6.2	63.2 ± 3.5
Race mean speed V_RCP_ (%)	73.4 ± 7.2	61.0 ± 6.5	72.4 ± 5.6
Race mean speed V_GET_ (%)	85.3 ± 6.5	73.0 ± 6.6	85.1 ± 7.0
Time in Zone 1 (min)	76.2 ± 52.2	124.4 ± 105.70	47.7 ± 32.50
Time in Zone 1 (%)	45.0 ± 30.0	50.0 ± 44.0	19.0 ± 15.0
Time in Zone 2 (min)	81.6 ± 50.4	66.4 ± 45.0	117.5 ± 43.5
Time in Zone 2 (%)	49.0 ± 31.0	26.0 ± 14.0	45.0 ± 15.0
Time in Zone 3 (min)	15.9 ± 11.3	89.9 ± 52.9	93.5 ± 53.8
Time in Zone 3 (%)	10.0 ± 7.0	9.1 ± 1.6	35.0 ± 21.0
luTRIMP_HR_ (a.u.)	202 ± 53	250 ± 35	563 ± 130

All values are presented as mean ± standard deviation

*HR* heart rate, HR_MAX_ (%): percentage of heart rate max, HR_RCP_ (%): percentage of heart rate at respiratory compensation point, HR_GET_ (%): percentage of heart rate at gas exchange threshold, V_MAX_ (%): percentage of velocity at maximal oxygen consumption, V_RCP_ (%): percentage of velocity at respiratory compensation point, V_GET_ (%): percentage of velocity at gas exchange threshold, luTRIMP_HR_: Lucia’s training impulse with heart rate in arbitrary unit (a.u.)

### Blood analysis

At T0, the cTnI values were above the URL in 11.8% of the participants (Table 4). At T1, cTnI increased by 19.6 ± 39.2 ng L^−1^ compared with that at T0 (*P* < 0.001, ES 0.67 *large*) (Fig. 1, Panel B), with 52.9% of participants having values above the URL (Table 4). At T2, cTnI values were not significantly different from those at T0 (*P* = 0.911), even though 5.9% of participants had values above the URL (Fig. 1, Panel B) (Table 4).

**Table 4:** Evaluation of participants' biochemical parameters against their respective reference values

	Reference values	T0		T1		T2	
		% below	% above	% below	% above	% below	% above
cTnI (ng L^−1^**)**	< 20		11.8		52.9		5.9
CK-MB (ng ml^−1^**)**	0–5		11.8		70.6		29.4
Pro-BNP (pg ml^−1^**)**	0–100						
Myoglobin (ng ml^−1^**)**	17–105	17.6			94.1		5.9
LDH (U L^−1^**)**	< 248		11.8		76.5		21.4
CREA (mg dl^−1^**)**	0.5–1.2				52.9		
eGFR (ml min^−1^**)**	90–120	17.6		88.2		21.4	

% below, percentage of subjects with marker level below the interval reference; % above, percentage of subjects with marker level above the interval reference or upper reference limits. Empty cells indicate 0.0%. *CREA* creatinine, *eGFR* estimated glomerular filtration rate, *cTnI* cardiac troponin I, *Pro-BNP* N-terminal proB-type natriuretic peptide, *LDH* lactate dehydrogenase, *CK-MB* creatine kinase-myocardial band

At T0, CK-MB levels were above the URL in 11.8% of the participants (Table 4). At T1, CK-MB increased by 3.41 ± 3.01 ng ml^−1^ compared with that at T0 (*P* = 0.002, ES 0.60 *large)* (Fig. 1, Panel C), with 70.6% of participants having values above the URL (Table 4). At T2, the CK-MB values were not significantly different from those at T0 (*P* > 0.999) (Fig. 1, Panel C), even though 25.0% of the participants had values above the URL (Table 4).

At T0, the Pro-BNP values were within the normal range (Fig. 1, Panel D) (Table 4). No significant differences were found for Pro-BNP at T1 and T2 compared with those at T0 (Fig. 1, Panel D) (Table 4).

At T0, myoglobin levels were below the reference limit in 17.6% of the participants (Table 4). At T1, myoglobin levels increased by 323.0 ± 228.0 ng ml^−1^ compared with those at T0, with 94.1% of participants having values above the URL (*P* < 0.001, ES 2.00 *large*) (Fig. 1, Panel E) (Table 4). At T2, myoglobin levels were not significantly different from those at T0 (*P* > 0.999) (Fig. 1, Panel E), even though 5.9% of the participants had values above the URL (Table 4).

At T0, 11.8% of the participants had LDH values above the URL (Table 4). At T1, LDH increased by 118.3 ± 64.0 U l^−1^ (*P* < 0.001, ES 1.69 *large)* (Fig. 1, Panel F) compared with that at T0, with 76.5% of participants having values above the URL (Table 4). At T2, LDH levels were not significantly different from those at T0 (*P* = 0.368) (Fig. 1, Panel F), even though 21.4% of the participants had values above the URL (Table 4).

At T0, CREA levels were within the normal range (Table 4). At T1, the CREA increased by 0.96 ± 0.10 U l^−1^ compared with that at T0 (*P* < 0.001, ES 2.04 *large)* (Fig. 1, Panel G), with 52.9% of participants having values above the URL (Table 4). At T2, the CREA values did not differ significantly from those at T0 (*P* = 0.080) (Fig. 1, Panel G).

At T0, the eGFR values were below the lower limits or reference values in 17.6% of the participants (Table 4). At T1, the eGFR decreased by -30.8 ± 10.4 ml min^−1^ compared with that at T0 (*P* < 0.001, ES 2.25 *large)* (Fig. 1, Panel H), with 88.2% of participants having values below the lower reference limits (Table 4). At T2, the eGFR values were significantly lower than those at T0 (P = 0.006, ES 0.55 *large*) and higher than those at T1 (*P* < 0.001, ES 1.87 *large*) (Fig. 1, Panel H), with 21.4% of the participants having values below the lower reference limits (Table 4).

### Correlation analysis

Correlation analyses between the changes (Δ) in the biochemical parameters and the baseline anthropometric data, physical fitness, and running-related variables are shown in Tables 5, 6, and 7, respectively.

**Table 5:** Correlations between the change (Δ) in biomarkers of cardiac, muscular, and renal function with the anthropometric characteristics of the participants at baseline (T0)

	ΔcTnI	ΔCK-MB	ΔMyoglobin	ΔLDH	ΔCREA	ΔeGFR
BM T0 (kg)	R = 0.461 P = 0.085	r = 0.067 P = 0.797	R = − 0.308 P = 0.228	r = − 0.149 P = 0.569	r = − 0.236 P = 0.361	r = 0.217 P = 0.404
FFM T0 (kg)	R = 0.554 P = 0.035	r = 0.133 P = 0.611	R = − 0.184 P = 0.479	r = 0.021 P = 0.936	r = − 0.067 P = 0.800	r = 0.061 P = 0.815
FM T0 (kg)	R = 0.029 P = 0.923	r = 0.031 P = 0.906	R = − 0.223 P = 0.388	r = − 0.284 P = 0.270	r = − 0.248 P = 0.338	r = 0.198 P = 0.447
FFM T0 (%)	R = 0.043 P = 0.883	r = 0.037 P = 0.886	R = 0.172 P = 0.509	r = 0.372 P = 0.142	r = 0.192 P = 0.461	r = − 0.119 P = 0.649
FM T0 (%)	R = − 0.043 P = 0.883	r = − 0.037 P = 0.886	R = − 0.172 P = 0.509	r = − 0.372 P = 0.142	r = − 0.192 P = 0.461	r = 0.119 P = 0.649
ΔBM T1-T0 (kg)	R = 0.223 P = 0.422	R = 0.174 P = 0.503	R = − 0.262 P = 0.308	r = 0.033 P = 0.900	R = 0.118 P = 0.651	R = − 0.020 P = 0.943

Δ = [(T1-T0)/T0]

*BM* body mass, *FFM* fat-free mass, *FM* fat mass, *CREA* creatinine, *eGFR* estimated glomerular filtration rate, *cTnI* cardiac troponin I, *LDH* lactate dehydrogenase, *CK-MB* creatine kinase-myocardial band

r value obtained with Pearson’s correlation coefficients

R value obtained with Spearman’s correlation coefficients (non‐normally distributed data).Bold text indicates a statistically significant correlation

**Table 6:** Correlations between the percentage change (Δ) in biomarkers of cardiac, muscular, and renal function with the physiological parameters of the participants at baseline (T0)

	ΔcTnI	ΔCK-MB	ΔMyoglobin	ΔLDH	ΔCREA	ΔeGFR (%)
V'O_2_max T0 (ml kg^−1^ min^−1^)	R = − 0.186 P = 0.507	r = 0.125 P = 0.632	R = 0.302 P = 0.239	r = 0.197 P = 0.445	r = − 0.204 P = 0.432	r = 0.191 P = 0.464
V'O_2_RCP T0 (ml kg^−1^ min^−1^)	R = − 0.161 P = 0.567	r = 0.275 P = 0.286	R = 0.441 P = 0.078	r = 0.357 P = 0.160	r = 0.160 P = 0.541	r = − 0.117 P = 0.656
V'O_2_GET T0 (ml kg^−1^ min^−1^)	R = − 0.364 P = 0.182	r = 0.119 P = 0.650	**R = 0.569 ** **P = 0.020**	r = 0.256 P = 0.321	r = 0.438 P = 0.079	r = − 0.404 P = 0.126
HRmax T0 (bpm)	R = − 0.404 P = 0.136	r = 0.299 P = 0.243	R = − 0.245 P = 0.341	r = − 0.200 P = 0.441	r = − 0.397 P = 0.115	r = 0.260 P = 0.313
HR_RCP_ T0 (bpm)	R = 0.458 P = 0.087	r = 0.033 P = 0.899	R = − 0.413 P = 0.100	r = − 0.137 P = 0.600	r = − 0.394 P = 0.117	r = − 0.013 P = 0.959
HR_GET_ T0 (bpm)	R = 0.050 P = 0.860	r = − 0.082 P = 0.754	R = − 0.167 P = 0.519	r = − 0.030 P = 0.911	r = − 0.035 P = 0.895	r = − 0.475 P = 0.054
V_MAX_ T0 (km h^−1^)	R = − 0.306 P = 0.265	r = 0.166 P = 0.523	**R = 0.624 ** **P = 0.009**	r = 0.142 P = 0.587	r = 0.184 P = 0.481	r = − 0.247 P = 0.338
V_RCP_ T0 (km h^−1^)	R = − 0.329 P = 0.232	r = 0.198 P = 0.447	**R = 0.602 ** **P = 0.012**	r = 0.097 P = 0.711	r = 0.206 P = 0.427	r = − 0.299 P = 0.243
V_GET_ T0 (km h^−1^)	R = − 0.310 P = 0.259	r = 0.233 P = 0.367	**R = 0.632 ** **P = 0.008**	r = 0.165 P = 0.526	r = 0.315 P = 0.218	r = − 0.381 P = 0.132

Δ = [(T1-T0)/T0]

V’O_2_max: maximal oxygen uptake, V’O_2_RCP: oxygens uptake at respiratory compensation point, V’O_2_GET: oxygen uptake at gas exchange threshold, *HRmax*: maximal heart rate, HR_RCP_: heart rate at respiratory compensation point, HR_GET_: heart rate at gas exchange threshold, V_MAX_: velocity at maximal oxygen uptake, V_RCP_: velocity at respiratory compensation point, V_GET_: velocity at gas exchange threshold, *cTnI*: cardiac troponin I, *CK-MB*: creatine kinase-myocardial band, *LDH*: lactate dehydrogenase, CREA: creatinine, *eGFR*: estimated glomerular filtration rate

r value obtained with Pearson’s correlation coefficients

R value obtained with Spearman’s correlation coefficients (non‐normally distributed data)

Bold text indicates a statistically significant correlation

**Table 7:** Correlations between the percentage change (Δ) in biomarkers of cardiac, muscular, and renal function with the exercise variables of the participants measured during the running competition

	ΔcTnI	ΔCK-MB	ΔMyoglobin	ΔLDH	ΔCREA	ΔeGFR
Time (min)	R = 0.081 P = 0.785	R = 0.480 P = 0.053	**R = 0.654 ** **P = 0.005**	R = 0.118 P = 0.653	R = 0.112 P = 0.668	R = − 0.042 P = 0.876
Race mean HR (bpm)	R = 0.022 P = 0.942	R = 0.120 P = 0.645	R = 0.287 P = 0.262	R = 0.248 P = 0.335	R = 0.331 P = 0.194	R = − 0.273 P = 0.288
Race mean HR_MAX_ (%)	R = − 0.231 P = 0.427	r = 0.034 P = 0.898	R = 0.188 P = 0.468	r = 0.292 P = 0.255	**r = 0.514 ** **P = 0.035**	r = − 0.452 P = 0.069
Race mean HR_RCP_ (%)	R = − 0.298 P = 0.300	r = 0.174 P = 0.503	R = 0.420 P = 0.095	r = 0.281 P = 0.274	**r = 0.544 ** **P = 0.024**	r = − 0.472 P = 0.056
Race mean HR_GET_ (%)	R = − 0.125 P = 0.302	r = 0.380 P = 0.133	**R = 0.546 ** **P = 0.026**	r = 0.311 P = 0.225	**r = 0.495 ** **P = 0.043**	r = − 0.457 P = 0.070
Race mean speed (km h^−1^)	R = − 0.341 P = 0.234	R = 0.159 P = 0.540	**R = 0.574 ** **P = 0.018**	R = − 0.022 P = 0.936	R = 0.197 P = 0.445	R = − 0.208 P = 0.421
Race mean speed V_MAX_ (%)	R = − 0.077 P = 0.797	r = − 0.137 P = 0.599	R = 0.244 P = 0.343	r = 0.234 P = 0.366	r = − 0.066 P = 0.799	r = 0.141 P = 0.589
Race mean speed V_RCP_ (%)	R = 0.015 P = 0.964	r = − 0.187 P = 0.473	R = 0.115 P = 0.660	r = 0.242 P = 0.349	r = − 0.144 P = 0.582	r = 0.265 P = 0.303
Race mean speed V_GET_ (%)	R = − 0.064 P = 0.832	r = − 0.292 P = 0.256	R = − 0.294 P = 0.250	r = 0.142 P = 0.586	r = -0.353 P = 0.165	r = 0.465 P = 0.069
Time in Zone 1 (s)	R = − 0.014 P = 0.974	r = − 0198 P = 0.479	R = − 0.443 P = 0.100	r = − 0.430 P = 0.110	r = − 0.435 P = 0.105	r = 0.320 P = 0.245
Time in Zone 1 (%)	R = 0.014 P = 0.974	r = − 0.328 P = 0.233	**R = −0.593 ** **P = 0.022**	r = − 0.470 P = 0.077	**r = − 0.518 ** **P = 0.048**	r = 0.437 P = 0.103
Time in Zone 2 (s)	R = − 0.217 P = 0.495	r = 0.033 P = 0.909	R = 0.271 P = 0.327	r = 0.452 P = 0.090	r = 0.217 P = 0.437	r = − 0.271 P = 0.329
Time in Zone 2 (%)	R = − 0.096 P = 0.754	r = -0.124 P = 0.659	R = 0.036 P = 0.900	r = 0.361 P = 0.187	r = -0.030 P = 0.915	r = 0.005 P = 0.984
Time in Zone 3 (s)	R = 0.018 P = 0.973	r = 0.308 P = 0.306	**R = 0.802 ** **P = 0.002**	r = 0.194 P = 0.504	**r = 0.800 ** **P = 0.002**	**r = − 0.659 ** **P = 0.017**
Time in Zone 3 (%)	R = − 0.377 P = 0.317	**r = 0.686 ** **P = 0.014**	**R = 0.862 ** **P = 0.001**	r = 0.071 P = 0.821	**r = 0.610 ** **P = 0.035**	**r = − 0.627 ** **P = 0.033**
luTRIMP_HR_ (a.u.)	R = 0.081 P = 0.785	R = 0.480 P = 0.053	**R = 0.654 ** **P = 0.005**	R = − 0.118 P = 0.653	R = 0.112 P = 0.668	R = − 0.042 P = 0.876

Δ = [(T1-T0)/T0]

HR_MAX_ (%): percentage of heart rate max, HR_RCP_ (%): percentage of heart rate at respiratory compensation point, HR_GET_ (%): percentage of heart rate at gas exchange threshold, V_MAX_ (%): percentage of velocity at maximal oxygen consumption, V_RCP_ (%): percentage of velocity at respiratory compensation point, V_GET_ (%): percentage of velocity at gas exchange threshold, luTRIMP_HR_: Lucia’s training impulse with heart rate. cTnI: cardiac troponin I, *CK-MB* creatine kinase-myocardial band, *LDH* lactate dehydrogenase, *CREA* creatinine, *eGFR* estimated glomerular filtration rate

r value obtained with Pearson’s correlation coefficients

R value obtained with Spearman’s correlation coefficients (non‐normally distributed data)

Bold text indicates a statistically significant correlation

**Fig. 2:**
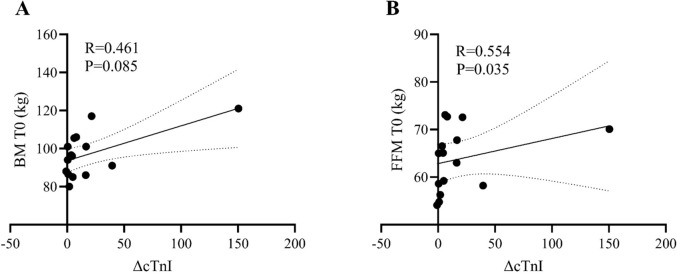
Correlations analysis between the change (Δ) in serum cTnI and the BM (**A**) and FFM (**B**) of the participants at baseline (T0). *Δ* = [(T1-T0)/T0]. *BM* body mass, *FFM* fat-free mass, *cTnI* cardiac troponin I. R value obtained with Spearman’s correlation coefficients (non‐normally distributed data)

The ∆cTnI was moderate but significantly correlated with the baseline FFM (kg) (R = 0.554, *P* = 0.035) (Fig. 2, Panel B).

**Fig. 3:**
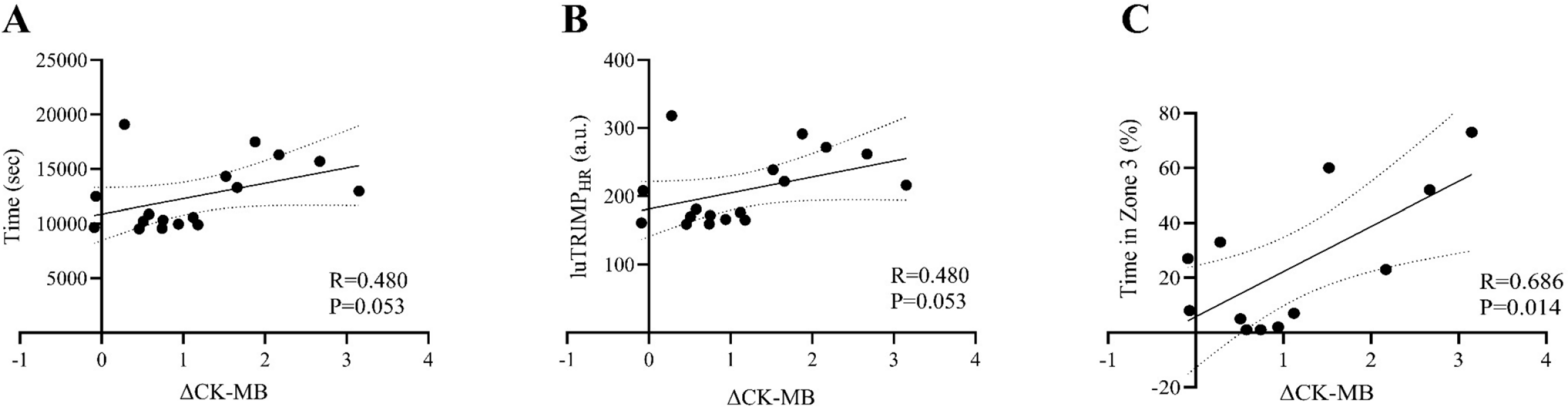
Correlations analysis between the change (Δ) in serum CK-MB and the running competition time (**A**), luTRIMP_HR_ (a.u.) (**B**), and the percentage (%) of time spent in zone 3 (**C**) of the participants measured during the running competition. *Δ* = [(T1-T0)/T0]. luTRIMPHR: Lucia’s training impulse with heart rate in arbitrary unit (a.u.), *CK-MB:* creatine kinase-myocardial band. R value obtained with Spearman’s correlation coefficients (non‐normally distributed data)

The ∆CK-MB was moderately correlated with the time spent in Zone 3 (%) during the marathon (*R* = 0.686; *P* = 0.014) (Fig. 3, Panel C).

**Fig. 4:**
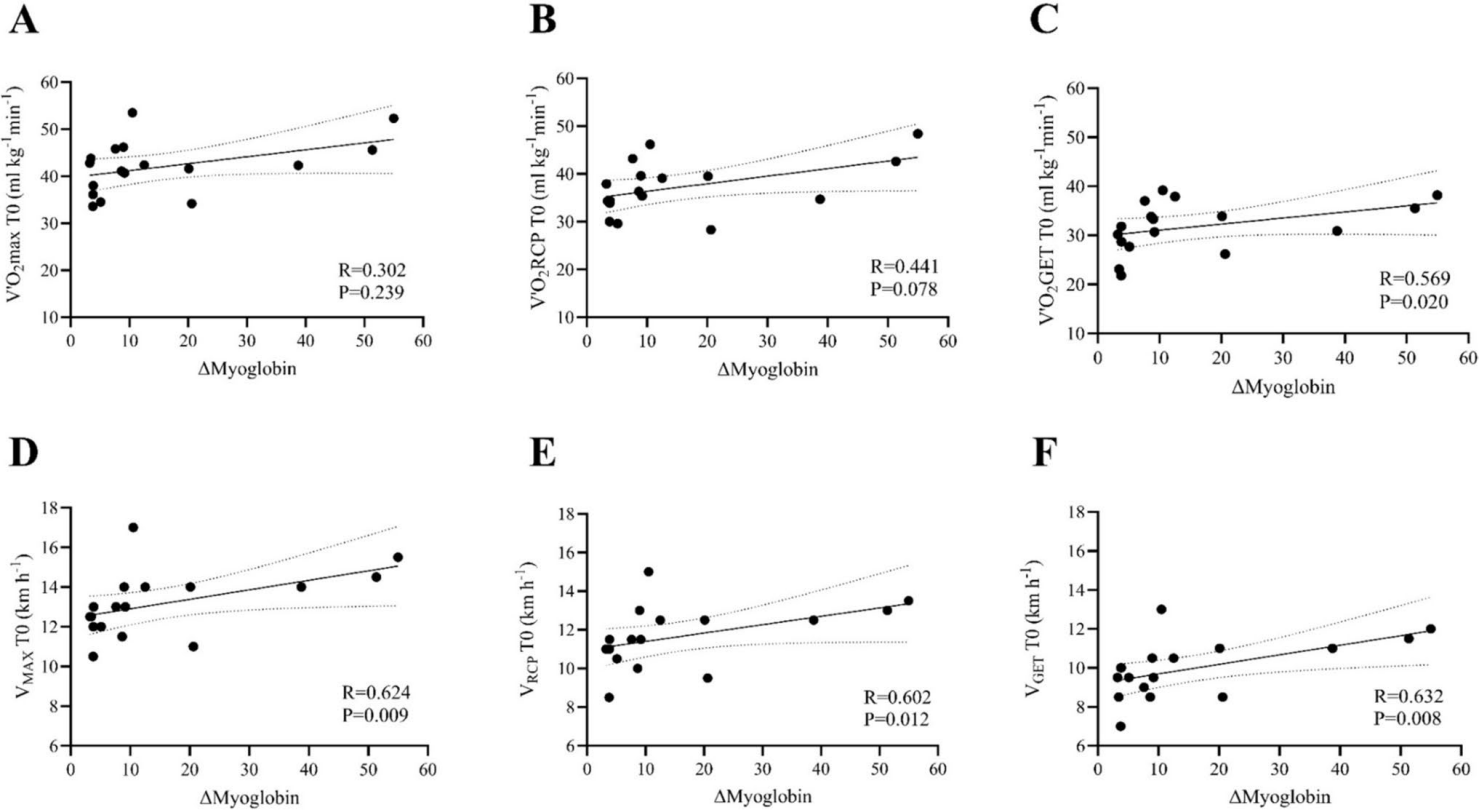
Correlations analysis between the change (Δ) in serum myoglobin and the V’O_2_max (**A**), VO_2_RCP (**B**), VO_2_GET (**C**), V_MAX_ (**D**), V_RCP_ (**E**), and V_GET_ (**F**) of the participants at baseline (T0). *Δ* = [(T1-T0)/T0]. V’O_2_max: maximal oxygen consumption, V’O_*2*_*RCP* oxygen consumption at respiratory compensation point, V’O_*2*_*GET* oxygen consumption at gas exchange threshold, V_*MAX*_* (km h*^*−1*^*): velocity at maximal oxygen consumption, V*_*RCP*_* (km h*^*−1*^*): velocity at respiratory compensation point, V*_*GET*_* (km h*.^*−1*^*): velocity at gas exchange threshold, R value obtained with Spearman’s correlation coefficients (non‐normally distributed data)*

**Fig. 5:**
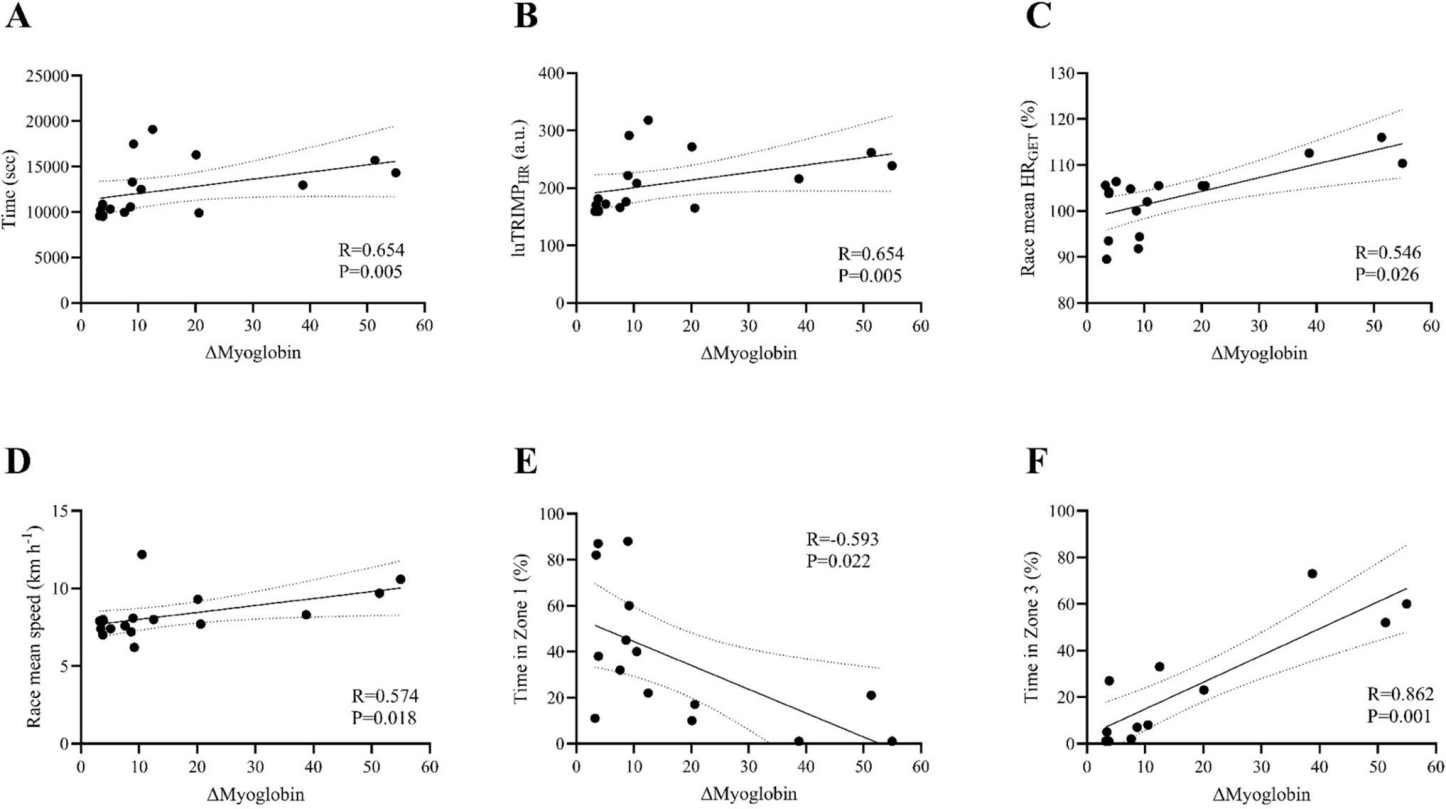
Correlations analysis between the change (Δ) in serum myoglobin and the running competition time (**A**), luTRIMPHR (a.u.) (**B**), the percentage (%) of race mean HR spent at HR_GET_ (**C**), race mean speed (**D**), % of time spent in zone 1 (**E**), and zone 3 (**F**) of the participants measured during the running competition. *Δ* = [(T1-T0)/T0]. luTRIMP_*HR*_*: Lucia’s training impulse with heart rate in arbitrary unit (a.u.), HR*_*GET*_* (%): percentage of heart rate at gas exchange threshold. R value obtained with Spearman’s correlation coefficients (non‐normally distributed data)*

The ∆myoglobin was moderately correlated with the baseline V’O_2_GET (*R* = 0.569; *P* < 0.020) (Fig. 4, Panel C), V_MAX_ (*R* = 0.624, *P* = 0.009) (Fig. 4, Panel D), V_RCP_ (*R* = 0.602; *P* < 0.012) (Fig. 3, Panel E), and V_GET_ (*R* = 0.632; *P* = 0.008) (Fig. 4, Panel F). In addition, ∆myoglobin was moderately correlated with marathon time (sec) (*R* = 0.654; *P* < 0.005) (Fig. 5, Panel A), luTRIMP_HR_ (*R* = 0.654, *P* = 0.004) (Fig. 5, Panel B), race mean HR_GET_ (%) (*R* = 0.546; *P* < 0.026) (Fig. 5, Panel C), and mean marathon speed (km h^−1^) (*R* = 0.574; *P* < 0.018) (Fig. 5, Panel D). In contrast, ∆myoglobin was inversely correlated with time spent in Zone 1 (%) during the marathon (*R* =  – 0.593; *P* = 0.022) (Fig. 5, Panel E) and strongly positively correlated with time spent in Zone 3 (%) during the marathon (*R* = 0.862; *P* = 0.001) (Fig. 5, Panel F).

**Fig. 6:**
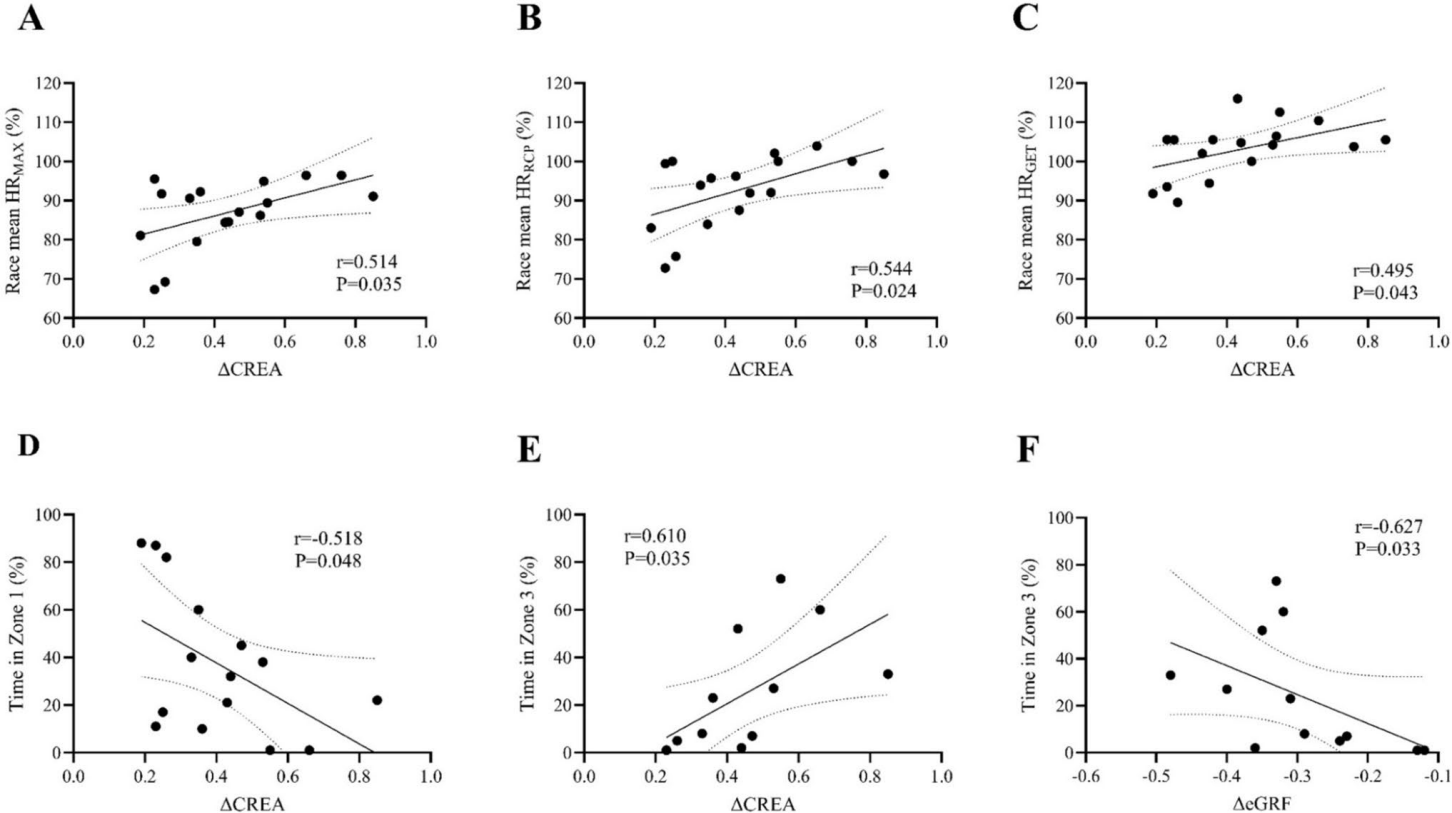
Correlations analysis between the change (Δ) in serum CREA and the running competition time (**A**), luTRIMP_HR_ (a.u.) (**B**), the percentage (%) of race mean HR spent at HR_GET_ (**C**), race mean speed (**D**), % of time spent in zone 1 (**E**) and zone 3 (**F**) of the participants measured during the running competition and the change (Δ) in serum eGRF and the % of time spent in zone 3 of the participants during the running competition (panel F). *Δ* = [(T1-T0)/T0]. HR_*MAX*_* (%): percentage of heart rate max, HR*_*RCP*_* (%): percentage of heart rate at respiratory compensation point, HR*_*GET*_* (%): percentage of heart rate at gas exchange threshold, CREA: creatinine, eGFR: estimated glomerular filtration rate. r value obtained with Pearson’s correlation coefficients. P:p-value*

The ∆CREA values were moderately correlated with the race mean HR_MAX_ (%) (*R* = 0.514; *P* = 0.035) (Fig. 6, Panel A), race mean HR_RCP_ (%) (*R* = 0.544, *P* = 0.024) (Fig. 6, Panel B), race mean HR_GET_ (*R* = 0.495; *P* = 0.043) (Fig. 6, Panel C), and time spent in Zone 3 (%) during the marathon (*R* = 0.610; *P* = 0.035) (Fig. 6, Panel E). In contrast, the ∆CREA values were inversely correlated with the time spent in Zone 1 (%) during the marathon (*R* =  – 0.518; *P* = 0.048) (Fig. 6, Panel D).

The ∆eGFR values were moderately inversely correlated with the time spent in Zone 3 (%) during the marathon (*R* =  – 0.627; *P* < 0.033) (Fig. 6, Panel F).

## Discussion

The main aim of the present study was to determine the changes in cardiac, muscular and renal biomarkers in healthy overweight or adults with obesity subjected to prolonged aerobic exercise through participation in a half-marathon, a 30-km run or a marathon based on the different cardiorespiratory fitness and performance levels they achieved at the end of the 24-week training programme (D’Alleva et al. 2023).

The main findings induced by running competition were (1) a significant reduction in BM not fully recovered 72 h after the race and a significant increase in the circulating levels of cTnI, CK-MB, myoglobin, LDH, CREA and eGFR, which returned to the reference limits 72 h after training; (2) ∆cTnI was correlated with the FFM baseline values; (3) ∆CK-MB was correlated with running-related variables; (4) ∆myoglobin was correlated with the baseline values of V’O_2_GET, velocity corresponding to ventilatory thresholds and V’O_2_max, and running-related variables; and (5) ∆CREA and ∆eGFR were correlated with training parameters.

In our study, we observed a transient increase in cardiac biomarkers (i.e. cTnI and CK-MB) after prolonged exercise. However, Pro-BNP increased after the marathon, albeit not significantly, similar to the results shown by Hu et al. (2023) in amateur runners. Instead, Traiperm et al. (2021), Sierra et al. (2019) and Hottenrott et al. (2016) reported a large increase in NT-Pro-BNP values after a marathon in elite and nonelite runners, which returned close to baseline 24 h after the race in all runners. However, despite the high NT-proBNP increase, no traumatic structural damage to the myocardium was detected by magnetic resonance (Traiperm et al. 2021).

The cTn is the most frequently measured cardiac marker for assessing the effects of prolonged exercise, and the majority of studies have shown an increase in cTnI after half-marathon, marathon or ultramarathon races in normal-weight subjects with different grades of training, as described in a recent meta-analysis (Rubio-Arias et al. 2021). In our study, post-race cTn levels increased successively after the race but returned to baseline 72 h after the race, which is in agreement with the findings of most studies in recreational athletes (e.g., a mean age of 38.6 years and a mean marathon time of 03 h and 30 min), who reported cTn levels in the reference value range within 96 h post-race (Bernat-Adell et al. 2019; Sierra et al. 2019). CK-MB followed the same trend as cTnI, increasing after the competition and then gradually decreasing until returning to initial values after 72 h post-race. These findings are in agreement with the findings of Hottenrott et al. (2016) in recreational athletes; however, CK-MB remained altered in recreational athletes even 15 days after the race in the study by Sierra et al. (2019). When we compare our data with those of studies conducted in recreational and well-trained runners during marathon and ultramarathon races on uphill, downhill or mixed terrain, we find similar values for the increase in markers of cardiac damage (e.g., cTnI, CK-MB and NT-Pro-BNP), which were six to ten times greater after the race (Koller et al. 2008; Da Ponte et al. 2018; Giovanelli et al. 2020). Therefore, the transient increase in cardiac damage biomarkers after prolonged exercise might be multifactorial. First, the response to extreme exercise in recreational and well-trained athletes could depend on genetic factors (Sierra et al. 2019) and nutritional status (Mielgo-Ayuso et al. 2020). However, genetic factors may not be the only players in this context. Indeed, the meta-regression of Rubio-Arias et al. (2021) reported a negative interaction between cTnI levels after a marathon and BMI in subjects with normal weight. Similarly, Shave et al. (2007) reported that in long-term aerobic events, the amount of cTnT released was related to BM. Nevertheless, Eijsvogels et al. (2012) reported that after a 30-, 40- and 50-km walk, the magnitude of absolute cTnI increase was comparable among lean, overweight, and individuals with obesity, suggesting that exercise intensity (i.e. as a percentage of HR_MAX_), rather than anthropometric factors, was the most important predictor of cTnI release after a long endurance event. In the present study, no relationship was observed between the ∆cTnI values and the baseline BM and FM. Nonetheless, a positive correlation was observed between ∆cTnI and baseline FFM, suggesting that cardiac work is greater in those with greater muscle mass. However, this finding should be confirmed in future studies. In addition, other studies have reported that the release of cTnI after long endurance events is influenced by other factors, such as exercise duration (Eijsvogels et al. 2015), energy expenditure during the race (Bernat-Adell et al. 2019) and training intensity [e.g., expressed as the mean HR, HR as a percentage of HR_MAX_ and HR_RCP_ (%) and marathon velocity expressed as V_RCP_ (%)] (Martínez-Navarro et al. 2020b; Rubio-Arias et al. 2021)]. In this study, we did not observe a correlation between ∆cTnI and running-related variables, as previously reported by Da Ponte et al. (2018).

With respect to CK-MB, the ∆CK-MB induced by the race was positive and significantly correlated with the time spent in Zone 3 (%) during training and displayed a trend towards significance when assessing its correlation with training time and luTRIMP_HR_. The latter suggests that the sustained increase in cardiac output over several hours of aerobic exercise, especially the time spent at high intensity, contributed to an increase in the myocardial workload, leading to the cytosolic release of biomarkers without true damage to myocytes (Hewing et al. 2015; Janssen et al. 2023).

In line with previous studies in experienced amateur runners, the present findings support the notion that prolonged aerobic exercise (i.e. over 90 min duration) also caused a transient increase in serum biomarkers of muscle damage (i.e. myoglobin and LDH) (Hottenrott et al. 2016; Del Coso et al. 2017), which returned to baseline levels within 72 h after the race. In addition, LDH levels increased after races within the marathon distance by an average of ~ 111 to  – 129 U l^−1^ (e.g., similar to our results) (Shin et al. 2016; Bernat-Adell et al. 2019), started to decrease 48 h after the race (Arakawa et al. 2016) and reached normalization 8 days after the race (Bernat-Adell et al. 2019). Instead, in the present study, LDH levels returned to baseline values within 72 h after the race compared with those reported in recreational participants in previous studies (Shin et al. 2016; Bernat-Adell et al. 2019). Several studies have shown that the release of myoglobin and LDH after long endurance competitions depends on the competition time (Bernat-Adell et al. 2019), running surface (e.g., uphill and downhill) (Giandolini et al. 2016; Da Ponte et al. 2018; Giovanelli et al. 2020), energy expenditure during the competition (Bernat-Adell et al. 2019), running intensity during the events (Hottenrott et al. 2016), heat and humidity (Gutiérrez-Vargas et al. 2018), and exercise-associated muscle cramps (Martínez-Navarro et al. 2020a). Compared with our results, we observed a similar increase in LDH after prolonged aerobic exercise in all the above conditions, whereas the increase in myoglobin was lower in our case than in running races with different surfaces. The latter could be partly due to the fact that compared to running on flat surfaces, running on uphill and downhill surfaces involves more work for type I and type II muscle fibres (Gottschall and Kram 2005), as concentric (e.g., uphill) (Gottschall and Kram 2005) (Gottschall and Kram 2005) and eccentric (e.g., downhill) (Giandolini et al. 2016) muscle contraction is more pronounced.

In the present study, no correlations between LDH and baseline anthropometric data, physical capacity, or running-related variables were detected. In contrast, ∆myoglobin levels were moderately correlated with baseline physiological parameters (i.e. V’O_2_GET, V_MAX_, V_RCP_ and V_GET_) and running-related variables [e.g., marathon time, luTRIMP_HR_, mean HR_GET_ (%), and mean marathon speed (km h^−1^)] and strongly positively correlated with time spent in Zone 3 (%) during the marathon. It is possible that a longer race time and greater average running intensity, despite different running distances, increase skeletal muscle damage and consequently increase the circulating levels of damage-related biomarkers after prolonged aerobic running (Gutiérrez-Vargas et al. 2018). In addition, muscular pH may decrease after a marathon, leading to fatigue with a decrease in force generation capacity, passive muscle tension and stiffness (Metzger and Moss 1990). To our knowledge, this is one of the first studies to compare biomarkers of muscular damage after long-term training and running-related parameters such as training load calculation (i.e. with luTRIMP_HR_) and the time spent in the three different HR zones during training in a cohort of participants with overweight or obesity.

Third, the transient increase in CREA levels and concomitant decrease in eGFR after prolonged aerobic exercise were observed as part of this study (i.e. over 90 min in duration), which suggested the onset of acute kidney injury in 35% of participants (Mehta et al. 2007; Shin et al. 2016). Previous studies have shown that serum CREA concentrations increase significantly by ~ 0.19–0.21 mg dL^−1^ after marathon and ultramarathon races in normal-weight recreational and well-trained athletes when different surfaces (e.g., uphill and downhill) are considered (Hewing et al. 2015; Poussel et al. 2020). In our study, the mean CREA increase was ~ 0.39 mg dL^−1^, and these differences could be due to the higher BMI of our participants. In addition, CREA levels returned to baseline within 72 h of the race and were within the reference range, which is consistent with previous studies in recreational and well-trained runners (Hewing et al. 2015; Poussel et al. 2020). For eGFR, previous studies in recreational and well-trained runners have shown that eGFR decreases after long-distance running (Hewing et al. 2015; Poussel et al. 2020) and returned to baseline at 24 h after the race (Panizo González et al. 2019). Instead, in this study, eGFR was lower even at 72 h post-race versus pre-race levels. This finding may be partly explained by the late arrival of intracellular toxic waste in the kidney, which was confirmed by the altered myoglobin and LDH levels, which were above the URL in 5.9% and 21.4% of participants, respectively (Panizo González et al. 2019). The physiological factors that contribute to the transient increase in renal biomarkers after prolonged exercise are multiple and contrasting. A few available studies in recreational and well-trained runners have shown that both the duration of the race and the main intensity of the race during a marathon or ultramarathon are more likely to lead to kidney damage (McCullough et al. 2011; Shin et al. 2016), whereas Hewing et al. (2015) reported a weak but significant negative correlation between lower eGFR values and the duration of the race. In addition to distance, some authors report an increase in markers of acute kidney damage after a marathon or ultramarathon on uphill, downhill or mixed terrain (Da Ponte et al. 2018; Giovanelli et al. 2020) due to increased concentric and eccentric muscle load and dehydration (Rojas-Valverde et al. 2021). However, there are conflicting results concerning the relationship between dehydration status and skeletal muscle damage after long-distance running with acute kidney injury (McCullough et al. 2011; Panizo González et al. 2019; Poussel et al. 2020).

In this study, no correlation was found between ∆CREA or ∆eGFR and baseline anthropometric data and physical capacity. Rather, ∆CREA values were moderately correlated with running-related variables (e.g., luTRIMP_HR_, mean HR of the race as a percentage of ventilatory thresholds and V’O_2_max) and strongly correlated with training time spent in Zone 3 (%). We also observed a negative correlation between the decrease in eGFR after a training session and the training time of participants in Zone 3 (%). It is possible that the higher average training intensity despite different training distances or training times (i.e. as shown by the negative correlation between CREA and race time in Zone 1) contributed to transient muscle damage that may lead to renal dysfunction due to glomerulus degeneration and reduced renal blood flow, resulting in reduced oxygen and energy supply and thus ischaemic damage to vascular endothelial cells (Brezis and Rosen 1995). Further studies are needed to better understand the mechanisms of acute kidney injury after long-distance running and to characterize the molecular mechanisms involved.

The present study has several limitations. First, although we detected changes in cardiac, muscular and renal biomarkers after prolonged aerobics, we did not consider a pre-exercise diet; food consumed during exercise; or hydration status before, during and after exercise. Although we measure the BM before and after the race, we do not know whether additional nutritional factors and hydration status could influence the results obtained. Second, although we observed a transient increase in markers of heart, muscle and kidney damage after exercise, we do not know if the affected organs were damaged. Future studies will also have to consider this last aspect. Third, our study involved a small sample size of healthy overweight or adults with obesity having different training levels (D’Alleva et al. 2023), and it was not possible to distinguish between the effects of the 3 different race distances (i.e. half-marathon, 30-km and marathon) on cardiac, muscular and renal biomarkers. Future studies are needed to further explore these aspects.

In conclusion, the results of this study provide a comprehensive overview of how cardiac, muscular, and renal damage occurs following prolonged running and 72 h post-exercise in a cohort of healthy adults with overweight or obesity. This is particularly important, as the number of recreational athletes, especially with overweight or Grade I obesity, competing in half-marathons or marathons has increased over the last 10 years. Therefore, when planning athletes' pre- and post-race training sessions, coaches should carefully consider the impact of baseline anthropometric data, physical capacity and running-related variables on markers of cardiac, muscular and renal damage.

## Data Availability

The corresponding author is available to share the primary data to those interested.

## Abbreviations

BIA: Bioelectrical impedance
BM: Body mass
BMI: Body mass index
CREA: Creatinine
CK-MB: N-Terminal creatine kinase-myocardial band
eGFR: Estimated glomerular filtration rate
FFM: Fat-free mass
FM: Fat mass
GET: Gas exchange threshold
GRAD: Graded exercise test
HR: Heart rate
LDH: Lactate dehydrogenase
NT-proBNP: Pro b-type natriuretic peptide
RCP: Respiratory compensation point
TL: Training load
TRIMP: Training impulse
URL: Upper reference limits
V’O_2_: Oxygen consumption
